# Automatic identification of suspicious bone metastatic lesions in bone scintigraphy using convolutional neural network

**DOI:** 10.1186/s12880-021-00662-9

**Published:** 2021-09-04

**Authors:** Yemei Liu, Pei Yang, Yong Pi, Lisha Jiang, Xiao Zhong, Junjun Cheng, Yongzhao Xiang, Jianan Wei, Lin Li, Zhang Yi, Huawei Cai, Zhen Zhao

**Affiliations:** 1 Laboratory of Clinical Nuclear Medicine, Department of Nuclear Medicine, West China Hospital, Sichuan University, No. 37 Guo Xue Alley, Chengdu, 610041 China; 2 Machine Intelligence Laboratory, College of Computer Science, Sichuan University, Chengdu, 610065 China

**Keywords:** Bone scintigraphy, Bone metastasis, Artificial intelligence, Convolutional neural network

## Abstract

**Background:**

We aimed to construct an artificial intelligence (AI) guided identification of suspicious bone metastatic lesions from the whole-body bone scintigraphy (WBS) images by convolutional neural networks (CNNs).

**Methods:**

We retrospectively collected the ^99m^Tc-MDP WBS images with confirmed bone lesions from 3352 patients with malignancy. 14,972 bone lesions were delineated manually by physicians and annotated as benign and malignant. The lesion-based differentiating performance of the proposed network was evaluated by fivefold cross validation, and compared with the other three popular CNN architectures for medical imaging. The average sensitivity, specificity, accuracy and the area under receiver operating characteristic curve (AUC) were calculated. To delve the outcomes of this study, we conducted subgroup analyses, including lesion burden number and tumor type for the classifying ability of the CNN.

**Results:**

In the fivefold cross validation, our proposed network reached the best average accuracy (81.23%) in identifying suspicious bone lesions compared with InceptionV3 (80.61%), VGG16 (81.13%) and DenseNet169 (76.71%). Additionally, the CNN model's lesion-based average sensitivity and specificity were 81.30% and 81.14%, respectively. Based on the lesion burden numbers of each image, the area under the receiver operating characteristic curve (AUC) was 0.847 in the few group (lesion number n ≤ 3), 0.838 in the medium group (n = 4–6), and 0.862 in the extensive group (n > 6). For the three major primary tumor types, the CNN-based lesion identifying AUC value was 0.870 for lung cancer, 0.900 for prostate cancer, and 0.899 for breast cancer.

**Conclusion:**

The CNN model suggests potential in identifying suspicious benign and malignant bone lesions from whole-body bone scintigraphic images.

**Supplementary Information:**

The online version contains supplementary material available at 10.1186/s12880-021-00662-9.

## Background

Bone metastasis commonly appears in the advanced stages of cancers [1–4]. It seriously affects the survival quality of patients due to the occurrence of adverse skeletal-related events [2, 5, 6]. The early diagnosis of bone metastasis is beneficial to make appropriate and timely treatment of metastatic bone disease, which can improve the quality of survival [7–10]. Even after the advent of single-photon emission computed tomography combined with computed tomography (SPECT/CT), whole-body bone scintigraphy (WBS) is a standard method to survey the existence and extent of bone metastasis [11]. However, the image resolution and the specificity of WBS are lacking [12]. And the interpretation of WBS is an experience-dependent work and the diagnostic agreement of inter-observer is not satisfactory [13].

Previously, we had proposed an automated diagnostic system of bone metastasis based on multi-view bone scans using an attention-augmented deep neural network [14, 15]. While it achieved considerable accuracy in the patient-based diagnosis from WBS images, a definitive diagnosis for suspicious bone metastatic lesions is still crucial for pragmatic decisions, such as precise bone biopsy, bone surgery and external beam radiotherapy [16]. Thus, a new artificial intelligence (AI) model with lesion-based diagnosis from the WBS image is more valuable for the clinic. Therefore, we fed a fully annotated WBS images dataset to construct a new AI model and evaluated its lesion-based performance in automatic diagnosing suspicious bone metastatic lesions.

## Methods

This retrospective single-center study was approved by the Institutional Ethics Committee of West China Hospital of Sichuan University. The written informed consent was waived from the Institutional Ethics Committee of West China Hospital of Sichuan University.

### Data resource

The WBS images of patients who were identified lung cancer, prostate cancer and breast cancer were retrieved from our hospital database within the period from Feb. 2012 to Apr. 2019. The WBS was performed using two gamma cameras (GE Discovery NM/CT 670 and Philips Precedence 16 SPECT/CT). The patient received 555 to 740 MBq of technetium-^99 m^ methylene diphosphonate (^99m^Tc-MDP; purchased from Syncor Pharmaceutical Co., Ltd, Chengdu, China) by intravenous injection, and the anterior and posterior views WBS images were obtained approximately 3 h post-injection. The gamma cameras were equipped with low-energy, high-resolution, parallel-hole collimators. The scan speed was 16–20 cm/min, and the matrix size was 512 × 1024. Energy peak was centered at 140 keV with 15% to 20% windows.

The visible bone lesion in WBS images was manually delineated by human experts and annotated into malignant and benign according to the following criteria [17, 18]:

Malignant: bone lesion with increased ^99m^Tc-MDP were identified as malignant (1) when computed tomography (CT), magnetic resonance imaging (MRI), positron emission tomography-computed tomography (PET/CT), etc. presented bone destruction; (2) when it appeared newly but couldn’t be ruled out as malignant in follow-up bone scan; (3) when it presented flare phenomenon; (4) when it enlarged and thickened significantly after at least 3 months follow-up.

Benign: bone lesion with increased ^99m^Tc-MDP were identified as benign (1) when CT, MRI and PET/CT, etc. demonstrated fracture, bone cyst, osteogeny, osteophyte, bone bridge, degenerative osteoarthrosis; (2) when it appeared around the bone joint; (3) when it confirmed as trauma.

**Fig. 1:**
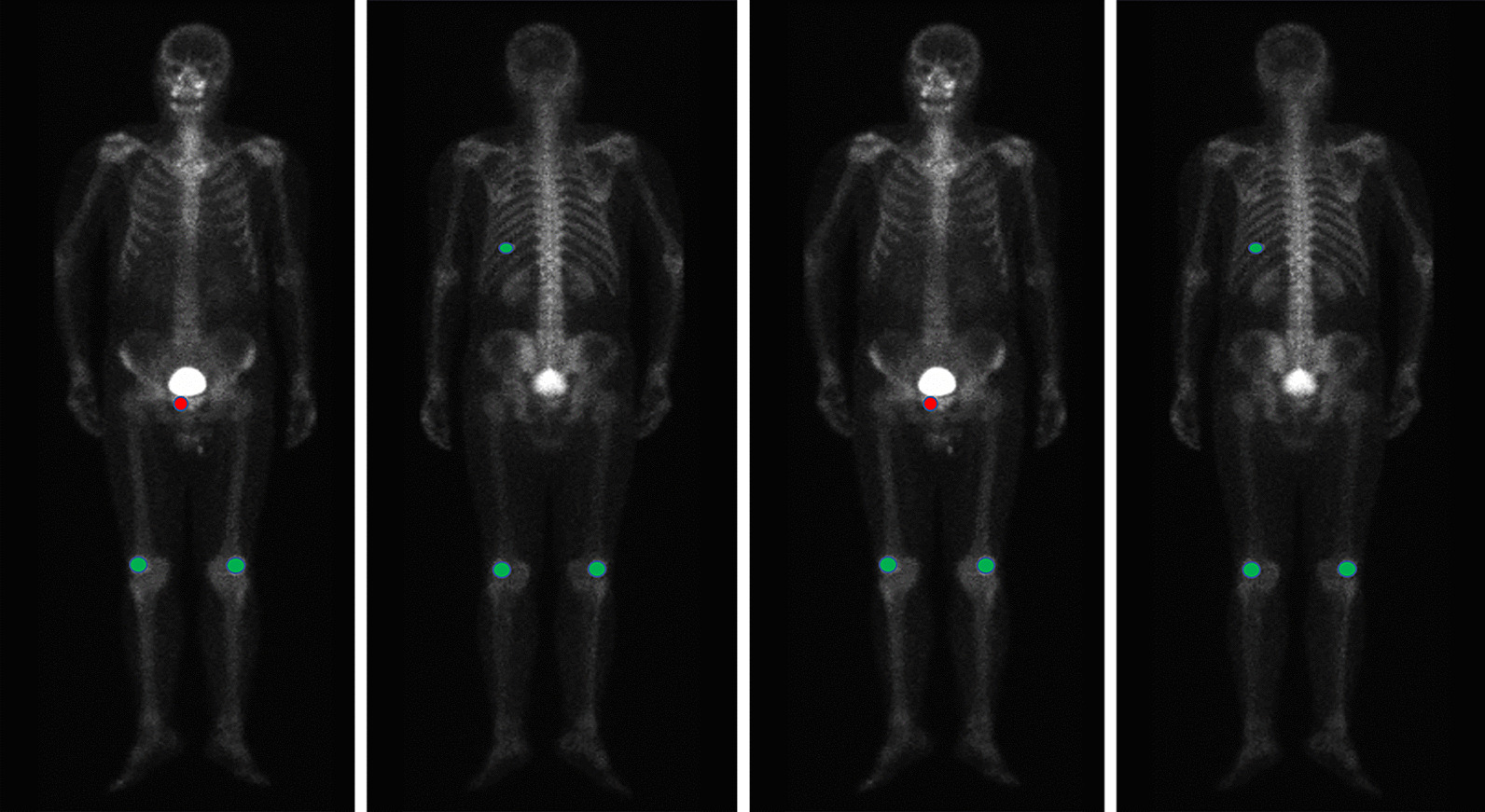
The diagram of manual annotation in WBS image. All visible bone lesions were delineated and annotated as benign and malignant. Red areas represent malignant lesions, while green areas represent benign lesions

The diagram of manual delineation and annotation was shown in Fig. 1. Additionally, the patient-based WBS image was assigned to malignant once a lesion was identified as malignant. Finally, from the 3352 patients, 14,972 visible bone lesions were identified as benign or malignant. According to the total number of lesions per WBS image [19], we divided all cases into three groups: few lesions group: 1–3 lesions; medium lesions group: 4–6 lesions; extensive lesions group: > 6 lesions.

### Model architecture

We implemented 2D CNN to automatically identification of bone metastatic lesions. Our network is based on the architecture of ResNet50 [20]. The CNN model was pre-trained on ImageNet, and fine-tuned on our own dataset. Before training the network, a pre-processing step was performed for data curation. The WBS and corresponding lesion mask were resized to 512 × 256. Considering the diagnosis of bone lesions was tremendously correlated to the location and burden extent, we stacked the full-sized images and the corresponding lesion mask on channel, instead of only inputting ROI of lesions. The data consisted of the original WBS image, the corresponding lesion mask and the qualitative of the lesion was used for CNN training. The fivefold cross validation was performed for evaluating the ability of the trained network model to achieve the qualitative task of bone scan lesions. Additionally, three state-of-the-art CNNs that included Inception V3 [21], VGG16 [22] and DenseNet169 [23] were compared with the proposed network.

**Fig. 2:**
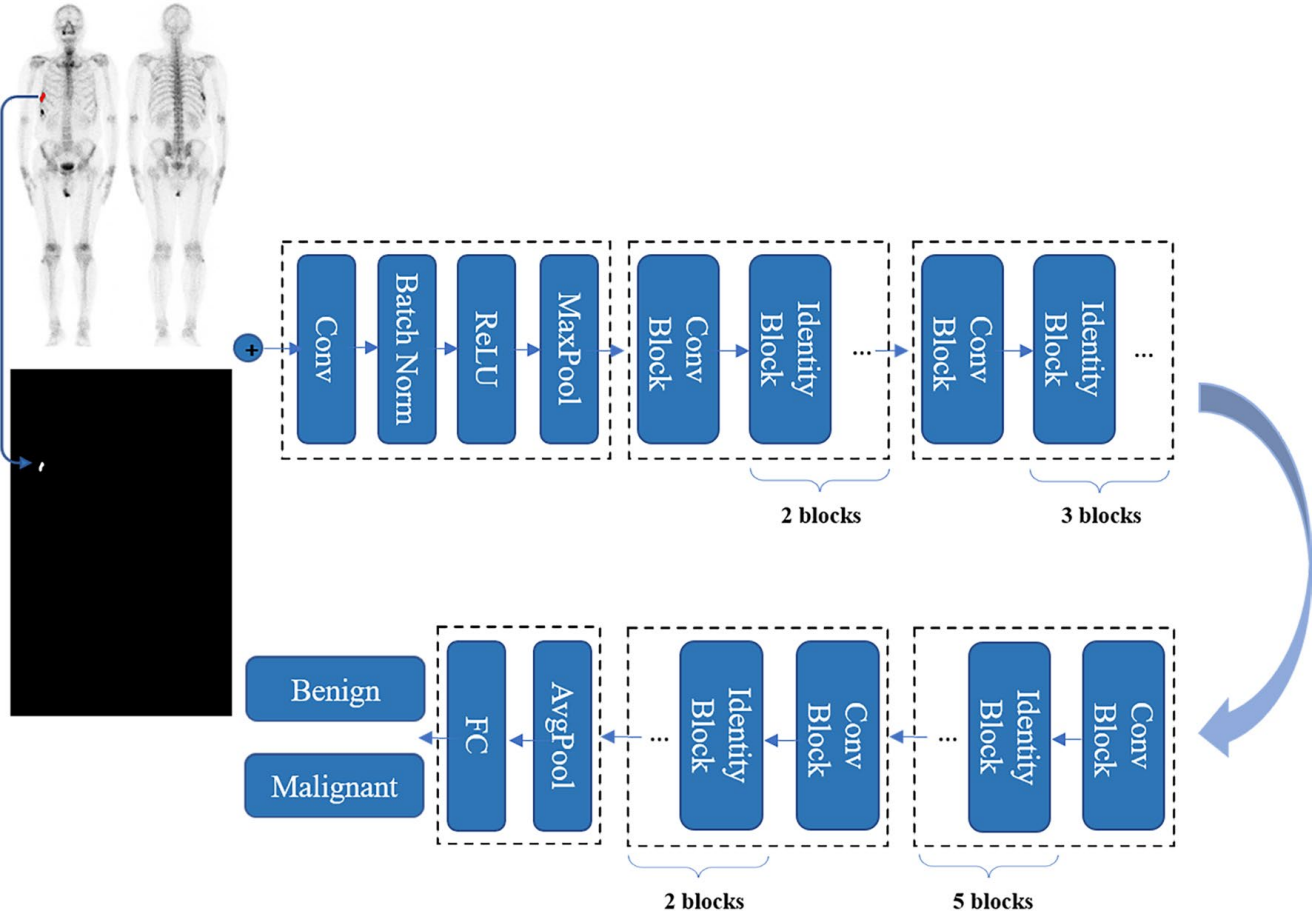
Architecture of convolutional neural network for AI model

The developed network was implemented using PyTorch [24], and trained using Adam [25] as the optimizer with a learning rate of 0.001 for 300 epochs. The mini-batch size was fixed 8. During the training process, random horizontal flipping with a probability of 0.5 was applied to the input to increase the diversity of the data. The detailed network architecture is shown in Fig. 2.

### Statistical analysis

The performance of AI was evaluated using diagnostic sensitivity, specificity, accuracy, positive predictive value (PPV), negative predictive value (NPV) and the area under the receiver operating characteristic curve (AUC). The Chi-square test was performed to compare differences in the AI performance between different number of lesions and different primary tumor types. The confusion matrix showed the numbers of true positive, true negative, false positive and false negative. All analyses were conducted using statistical software SPSS22.0 (SPSS Inc, Chicago, Illinois, USA). *P* values less than 0.05 were considered statistically significant.

## Results

### Baseline characteristics of patients

3352 cancer patients (Age: 61.61 ± 12.69y; Gender: 1758 males and 1594 females) were retrospectively included in the study and 43.85% of all patients presented bone metastasis. A total of 14,972 visible bone lesions were recognized in all WBS images and 51.23% of them were identified metastasis. The lesion-based metastasis rate was 50.13% in lung cancer, 57.39% in prostate cancer, and 44.61% in breast cancer, respectively. The detailed information was listed in Table 1.

**Table 1:** The summary of patient-based and lesion-based analysis in all WBS images

	Lung cancer	Prostate cancer	Breast cancer	Total
*Patient-based*				
Total	1253	1017	1082	3352
Malignant	567	466	437	1470
Benign	686	551	645	1882
Metastasis rate	45.25%	45.82%	40.39%	43.85%
*Lesion-based*				
Total	4937	5623	4412	14,972
Malignant	2475	3227	1968	7670
Benign	2462	2396	2444	6812
Metastasis rate	50.13%	57.39%	44.61%	51.23%

### The performance of the proposed network

After fivefold cross validation, the CNN model demonstrated an average sensitivity, specificity, accuracy, PPV and NPV for all visible bone lesions were 81.30%, 81.14%, 81.23%, 81.89% and 80.61%, respectively. When compared with the other three start-of-art CNNs, our proposed network achieved the best accuracy in identification the bone lesions at bone scintigraphy (Tables 2, 3).

**Table 2:** The fivefold cross validation results of the proposed network

	Fold 1	Fold 2	Fold 3	Fold 4	Fold 5
Number of patients (benign/malignant)	669 (376/293)	669 (376/293)	671 (376/295)	669 (376/293)	674 (378/296)
Number of lesions (benign/malignant)	2986 (1505/1481)	2952 (1389/1563)	3084 (1468/1616)	2897 (1475/1422)	3053 (1465/1588)
Sensitivity	83.19	83.11	79.89	76.79	83.5
Specificity	78.07	82.58	82.7	82.78	79.59
Accuracy	80.61	82.86	81.23	79.84	81.62
PPV	78.87	84.3	83.56	81.13	81.6
NPV	82.51	81.29	78.88	78.72	81.65

**Table 3:** The comparison of the proposed network and other three networks

	Sensitivity	Specificity	Accuracy	PPV	NPV
Our model	81.30	81.14	81.23	81.89	80.61
InceptionV3	77.29	84.02	80.61	83.53	78.00
VGG16	78.73	83.51	81.13	83.39	79.14
DenseNet169	67.90	85.73	76.71	83.17	72.16

### Subgroup analysis of proposed network

**Fig. 3:**
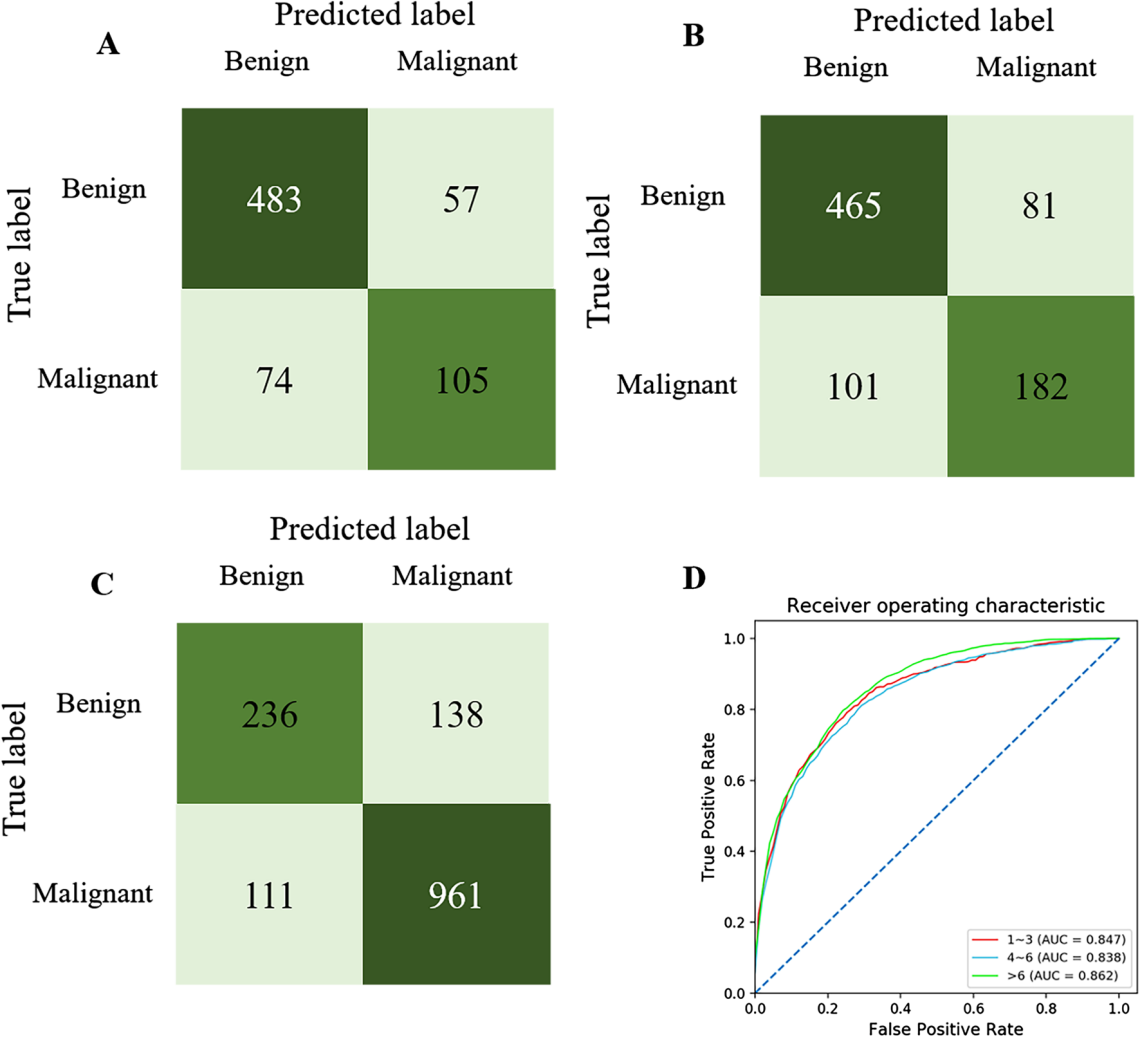
The confusion matrix of few lesions group (**A**), medium lesions group (**B**), extensive lesions group (**C**). The ROC of the three groups in the lesion-based diagnosis (**D**)

Based on the number of lesions per image, we found that the AI model reached the highest sensitivity (89.56%, *P* < 0.001), accuracy (82.79%, *P* = 0.018) and PPV (87.37%, *P* < 0.001) in the extensive lesions group as shown in Table 4. Whereas, the highest specificity (89.41%, *P* < 0.001) and NPV (86.76%, *P* < 0.001) of the AI model were captured in few lesions group. We also calculated the AUC to evaluate the diagnostic performance of the AI model, which was 0.847 in the few lesions group, 0.838 in the medium lesions group, and 0.862 in the extensive lesions group. And the confusion matrix directly demonstrated the true labels and predicted labels in the three groups (Fig. 3).

**Table 4:** The lesion-based diagnostic performance of AI model in testing cohort and the comparison of the AI performance among few, medium and extensive lesion groups

	Group for number of lesions			χ^2^	*P* value
	Few	Medium	Extensive		
Sensitivity	58.63	64.34	89.56	163.41	< 0.001
Specificity	89.41	85.24	62.85	108.69	< 0.001
Accuracy	81.78	78.03	82.79	8.06	0.018
PPV	64.89	69.67	87.37	83.70	< 0.001
NPV	86.76	82.04	67.93	49.24	< 0.001

Chi-square test was performed to compare the performance of AI model among different groups of the number of lesions. Few lesions group: 1–3 lesions per image; Medium lesions group: 4–6 lesions per image; Extensive lesions group: > 6 lesions per image

**Fig. 4:**
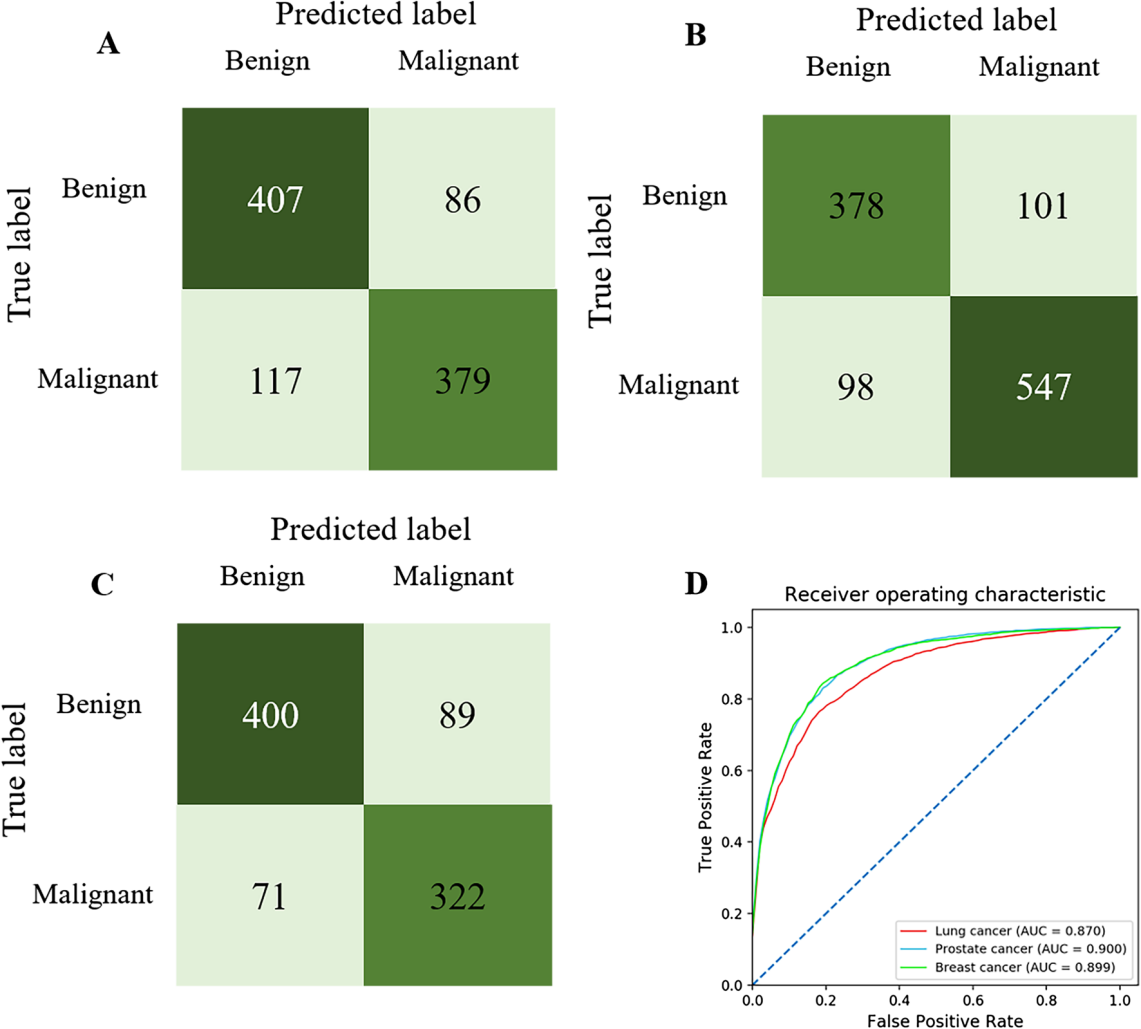
The confusion matrix of lung cancer group (**A**), prostate cancer group (**B**), breast cancer group (**C**). The ROC of the three groups in the lesion-based diagnosis (**D**)

The detailed results based on the primary tumor types were shown in Table 5, the results demonstrated the highest diagnostic sensitivity (84.66%, *P* = 0.002) in the prostate cancer group. Albeit slightly higher accuracy (82.30%) in the prostate cancer group, there was no statistical significance (*P* = 0.209) comparing with the lung cancer group (79.40%) and breast cancer group (81.82%). The specificity in lung cancer (82.52%), prostate cancer (79.07%) and breast cancer (81.78%) group also did not indicate statistical significance between each other (*P* = 0.354). Furthermore, the AUC was 0.870 for lung cancer, 0.900 for prostate cancer, 0.899 for breast cancer. The confusion matrix directly demonstrated the true labels and predicted labels in the three groups (Fig. 4).

**Table 5:** The lesion-based diagnostic performance of AI model in the testing cohort and the comparison of the AI performance among lung, prostate and breast cancers

	Group for primary tumor types			χ^2^	*P* value
	Lung cancer	Prostate cancer	Breast cancer		
Sensitivity	76.16	84.66	81.65	12.88	0.002
Specificity	82.52	79.07	81.78	2.08	0.354
Accuracy	79.40	82.30	81.82	3.13	0.209
PPV	81.12	84.43	78.33	6.52	0.038
NPV	77.70	79.52	85.05	8.85	0.012

Chi-square test was performed to compare the performance of AI model among different tumor types

Additionally, we also evaluated the lesion-based diagnostic performance of the AI model according to the different number of lesions per image (few, medium and extensive lesions group) in lung cancer, prostate cancer and breast cancer, respectively. The results were supported as Additional file 1: Table 1 and Additional file 2: Figs. 1, 2, and 3.

## Discussion

The definitive identification of abnormal bone lesions is beneficial to proper personalized treatment and subserves the patients who were suffering from advanced malignant cancers [26]. However, the precise differentiation of suspicious bone lesions is still tricky based on the WBS images only [27]. In light of the superiority of artificial intelligence in image feature extraction and big data analysis, we developed a new AI model using the 2D CNN to explore the potential for automatically definitive identification of suspicious bone metastatic lesions from WBS images.

In general, our AI model achieved moderate performance in the identification of suspicious bone lesions with a sensitivity of 81.30% and specificity of 81.14%. We found that AI indicated significantly higher accuracy in the extensive-lesions group (n > 6, accuracy = 82.79%) than that in the few-lesions group (n ≤ 3, accuracy = 81.78%, *p* < 0.05) and medium-lesions group (n = 4–6, accuracy = 78.03%, *p* < 0.05), this might be beneficial from the deep neural network which imitating human thinking model. Originally, classification of every single lesion is judged independently, regardless of the other lesions that appeared in the same image. However, nuclear medicine physicians usually take other lesions and additional cues into account when determining one single lesion itself. For example, an isolated lesion without other nearby lesions would be more difficult to assert benign or malignant, while multiple lesions that occur within a narrow region would be more likely malignant. We input corresponding lesion masks to the CNN and take the whole WBS image into account, and this might be a possible reason for the improved accuracy of the extensive-lesions group.

Previous studies also reported AI for bone lesion identification from WBS images. The authors used a ladder network to pre-train a nerual network with an unlabeled dataset [28]. On the metastasis classification task, It reached a sensitivity of 0.657 and a specificity of 0.857. Another similar study also build a model to detect and identify bone metastasis from bone scintigraphy images through negative mining, pre-training, the convolutional neural network, and deep learning [29]. The mean lesion-based sensitivity and precision rates for bone metastasis classification were 0.72 and 0.90, respectively. In our study, the lesion-based sensitivity, specificity and precision values for metastasis classification were 0.813, 0.811 and 0.819, respectively. It is difficult to compare the difference of algorithms, all studies have used in-house datasets of a gold standard and these datasets were not open. We were not able to try other datasets using our algorithm. Therefore, the performances reported by other researchers can only be used as references, rather than for objective comparison. It is worth mentioning that the aforementioned AI was focused on the chest image instead of the whole body. This strategy excluded the influence from keen osteoarthritis, degenerative changes of lumbar/cervical vertebrae, but it was limited to analyzing the metastases in other regions such as the pelvis, sacrum, iliac joints and other distant lesions. Addittionaly, we stacked the WBS and the corresponding lesion mask in channel and input it into the network. Thus, this CNN approach could select any suspicious bone lesion that needs to be input manually and obviate missed lesion detection and wrong lesion detection.

Three common kinds of primary cancers were investigated in this study. The different sensitivity among primary cancer types seemed to be affiliated to osteoblastic and osteolytic activity. The highest sensitivity appeared in the prostate cancer group and it is consistent with other former studies [17]. The probable reason is due to the typical osteoblastic metastasis principally in prostate cancer, though it is also associated with the osteoclastic process and bone resorption [30]. On the other hand, lung cancer and breast cancer group showed more significant osteolytic changes and corresponding mild radioactivity in lesions [31, 32].

Generally, our AI model achieved a moderate accuracy, sensitivity and specificity in the lesion-based diagnosis of WBS images, the false-positive lesions and false-negative lesions still could not be avoided. It is limited to the substantive character and specificity of ^99m^Tc-MDP imaging technology. Most pathological bone conditions, whether of infectious, traumatic, neoplastic or other origin could demonstrate as an increased radioactive signal in WBS images [33]. There are still several limitations in the current study. Firstly, since it is impossible to obtain the pathological result of each lesion, we made the “gold labels” based on the patients’ medical records, the follow-up bone scans, CT, MRI, PET/CT images, etc., which may not be totally correct for every lesion. Secondly, the labeled lesions on WBS images were all visible, which means only the “hotspots” were included, whereas some “cold lesions” were missed. Then, at present, this AI model was constructed by those non-quantitative images, the indraught of anatomical localization parameter and quantitative index might further improve the property, all of which would be paid attention in our future studies. Even though the AI model is not always correct, it still can be used by nuclear medicine physicians for assisting the bone lesions analysis and the final interpretation of an examination, especially for the patients who could not be performed SPECT/CT timely due to the poverty of resource devices.

## Conclusions

The AI model based on CNN reached a moderate lesion-based performance in the diagnosis of suspicious bone metastatic lesions from WBS images. Even though the AI model is not always correct, it could serve as an effective auxiliary tool for diagnosis and guidance in patients with suspicious bone metastatic lesions in daily clinical practice.

## Supplementary Information

**Additional file 1**. The lesion-based diagnostic performance of the AI model according to the different number of lesions per image (few, medium and extensive lesions group) in lung cancer, prostate cancer and breast cancer.

**Additional file 2**: Fig. S1. The confusion matrix of few lesions group (A), medium lesions group (B), extensive lesions group (C) in lung cancer group. The ROC of the three groups in the lesion-based diagnosis (D).

**Additional file 3**: Fig. S2. The confusion matrix of few lesions group (A), medium lesions group (B), extensive lesions group (C) in prostate cancer group. The ROC of the three groups in the lesion-based diagnosis (D).

**Additional file 4**: Fig. S3. The confusion matrix of few lesions group (A), medium lesions group (B), extensive lesions group (C) in breast cancer group. The ROC of the three groups in the lesion-based diagnosis (D).

## Data Availability

The datasets generated and analyzed during the current study are not publicly available but available from the corresponding author upon reasonable request.

## Abbreviations

AI: Artificial intelligence
WBS: Whole-body bone scintigraphy
AUC: Area under receiver operating characteristic curve
SPECT/CT: Single-photon emission computed tomography combined with computed tomography
CT: Computed tomography
MRI: Magnetic resonance imaging
PET/CT: Positron emission tomography-computed tomography
CNN: Convolution neural networks
PPV: Positive predictive value
NPV: Negative predictive value
