# Supplementary material for: Automatic identification of suspicious bone metastatic lesions in bone scintigraphy using convolutional neural network
Source: BMC Med Imaging. 2021 Sep 4;21:131. doi: 10.1186/s12880-021-00662-9 (PMC8417997; doi:10.1186/s12880-021-00662-9)
Supplement: Supplementary file 1 — Additional file 1. The lesion-based diagnostic performance of the AI model according to the different number of lesions per image (few, medium and extensive lesions group) in lung cancer, prostate cancer and breast cancer. [file 12880_2021_662_MOESM1_ESM.docx]

**Supplementary Materials**

**Supplementary Table1.**

| Primary tumor type |  | Group for number of lesions | | | | χ2 | P value |
| --- | --- | --- | --- | --- | --- | --- | --- |
|  |  | Few | Medium | Extensive | |  |  |
| Lung cancer | Sensitivity | 57.41 | 56.89 | 88.14 | 63.73 | | ＜0.001 |
|  | Specificity | 89.48 | 83.19 | 66.96 | 23.37 | | ＜0.001 |
|  | Accuracy | 80.34 | 73.48 | 82.54 | 10.12 | | 0.006 |
|  | PPV | 68.92 | 66.58 | 87.90 | 31.32 | | ＜0.001 |
|  | NPV | 84.06 | 76.48 | 66.41 | 12.51 | | 0.002 |
|  |  |  |  |  |  | |  |
| Prostate cancer | Sensitivity | 58.13 | 68.94 | 89.43 | 49.42 | | ＜0.001 |
|  | Specificity | 90.94 | 85.93 | 57.59 | 54.40 | | ＜0.001 |
|  | Accuracy | 84.10 | 79.83 | 82.43 | 1.26 | | 0.532 |
|  | PPV | 63.65 | 73.66 | 87.75 | 26.74 | | ＜0.001 |
|  | NPV | 89.05 | 83.56 | 62.03 | 33.55 | | ＜0.001 |
|  |  |  |  |  |  | |  |
| Breast cancer | Sensitivity | 60.58 | 68.05 | 90.41 | 40.19 | | ＜0.001 |
|  | Specificity | 88.03 | 86.56 | 63.93 | 30.01 | | ＜0.001 |
|  | Accuracy | 81.57 | 80.99 | 82.78 | 0.35 | | 0.838 |
|  | PPV | 61.68 | 69.06 | 85.05 | 22.00 | | ＜0.001 |
|  | NPV | 88.02 | 86.39 | 77.72 | 7.44 | | 0.024 |

Chi-square test was performed to compare the performance of AI model among different groups of number of lesions. Few lesions group: 1-3 lesions per image; Medium lesions group: 4-6 lesions per image; Extensive lesions group: >6 lesions per image.
