# Supplementary figures and images for: Automatic identification of suspicious bone metastatic lesions in bone scintigraphy using convolutional neural network
Source: BMC Med Imaging. 2021 Sep 4;21:131. doi: 10.1186/s12880-021-00662-9 (PMC8417997; doi:10.1186/s12880-021-00662-9)

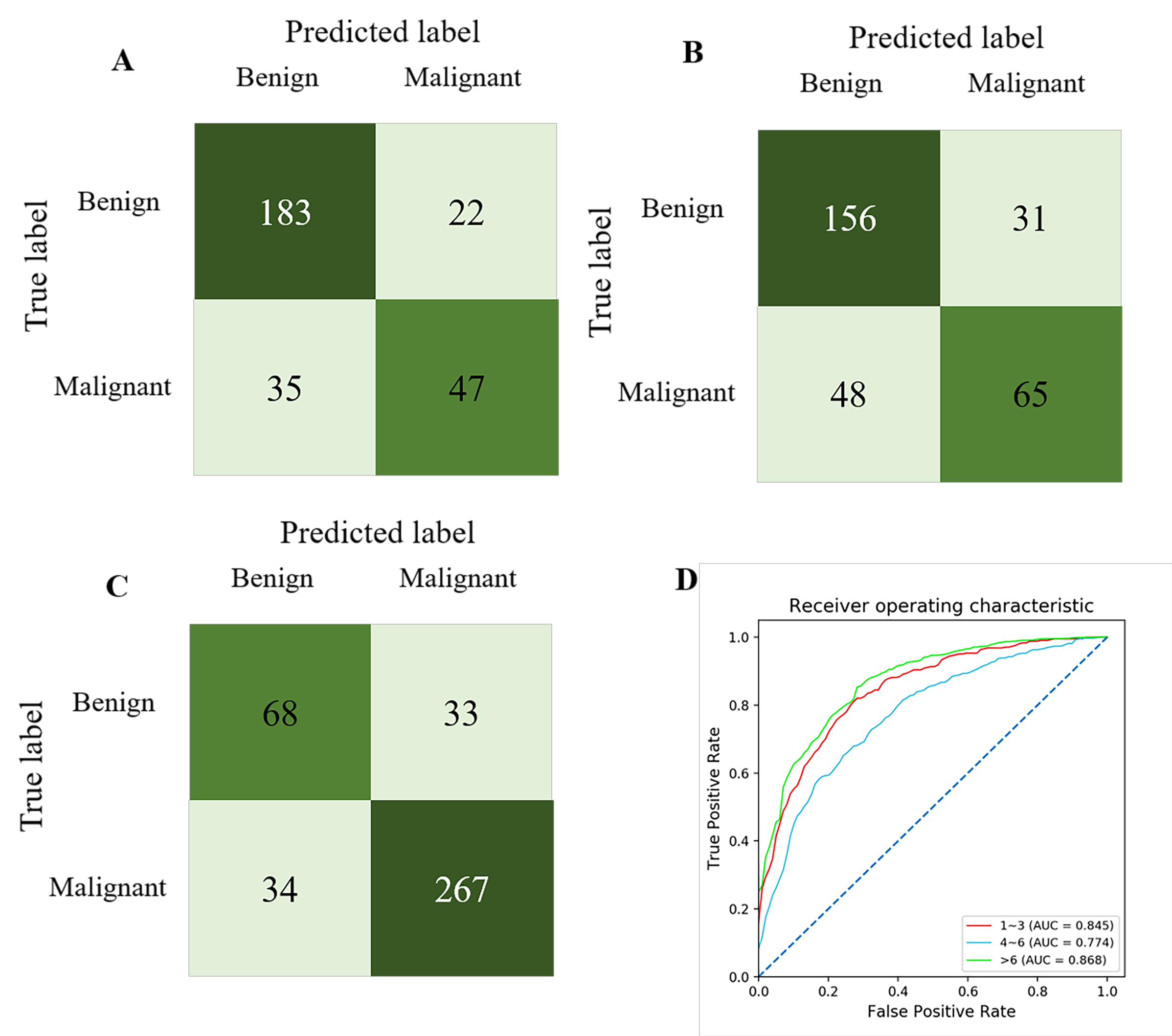

Supplement: Supplementary file 2 — Additional file 2: Fig. S1. The confusion matrix of few lesions group (A), medium lesions group (B), extensive lesions group (C) in lung cancer group. The ROC of the three groups in the lesion-based diagnosis (D). [file 12880_2021_662_MOESM2_ESM.tif]

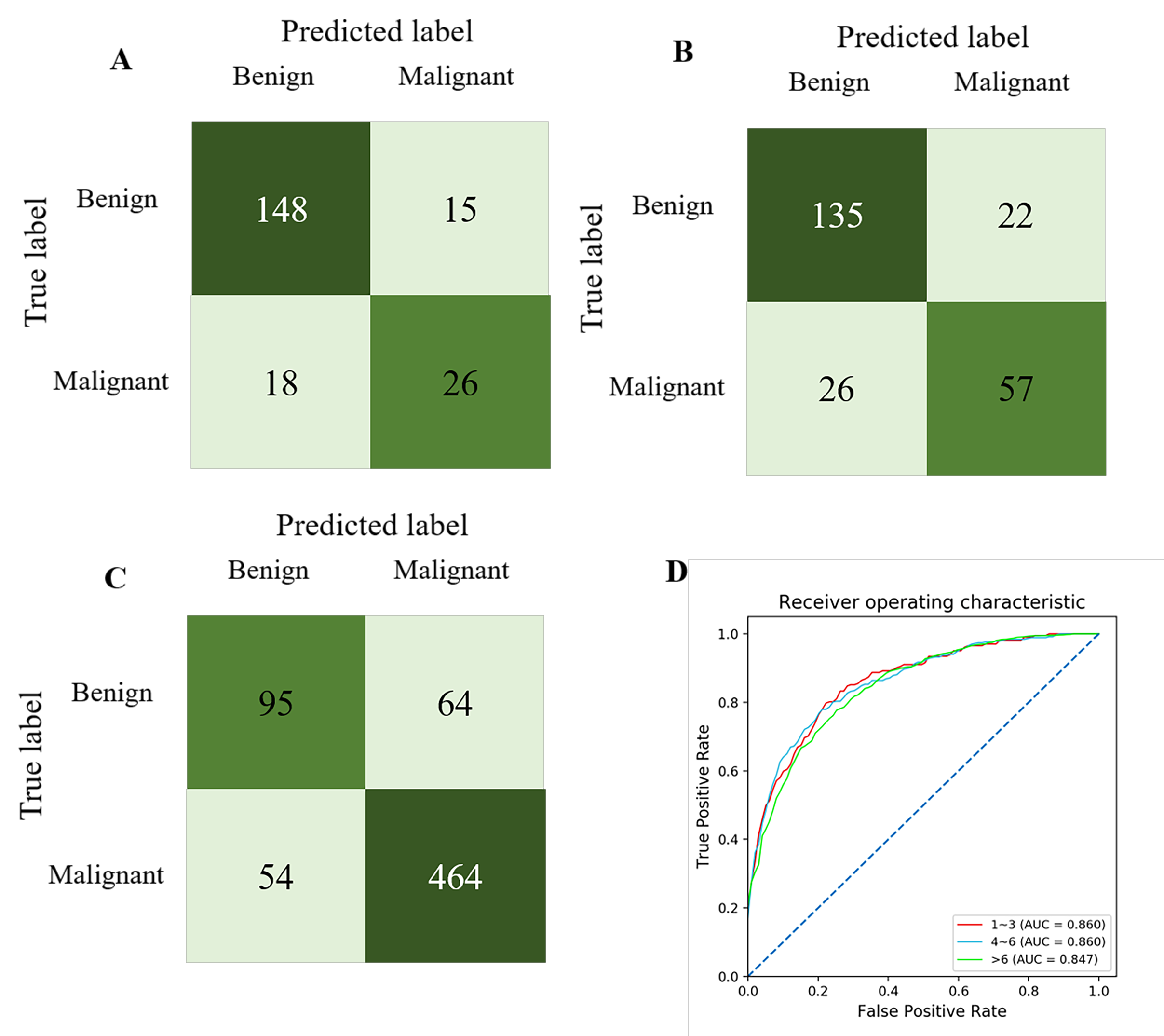

Supplement: Supplementary file 3 — Additional file 3: Fig. S2. The confusion matrix of few lesions group (A), medium lesions group (B), extensive lesions group (C) in prostate cancer group. The ROC of the three groups in the lesion-based diagnosis (D). [file 12880_2021_662_MOESM3_ESM.tif]

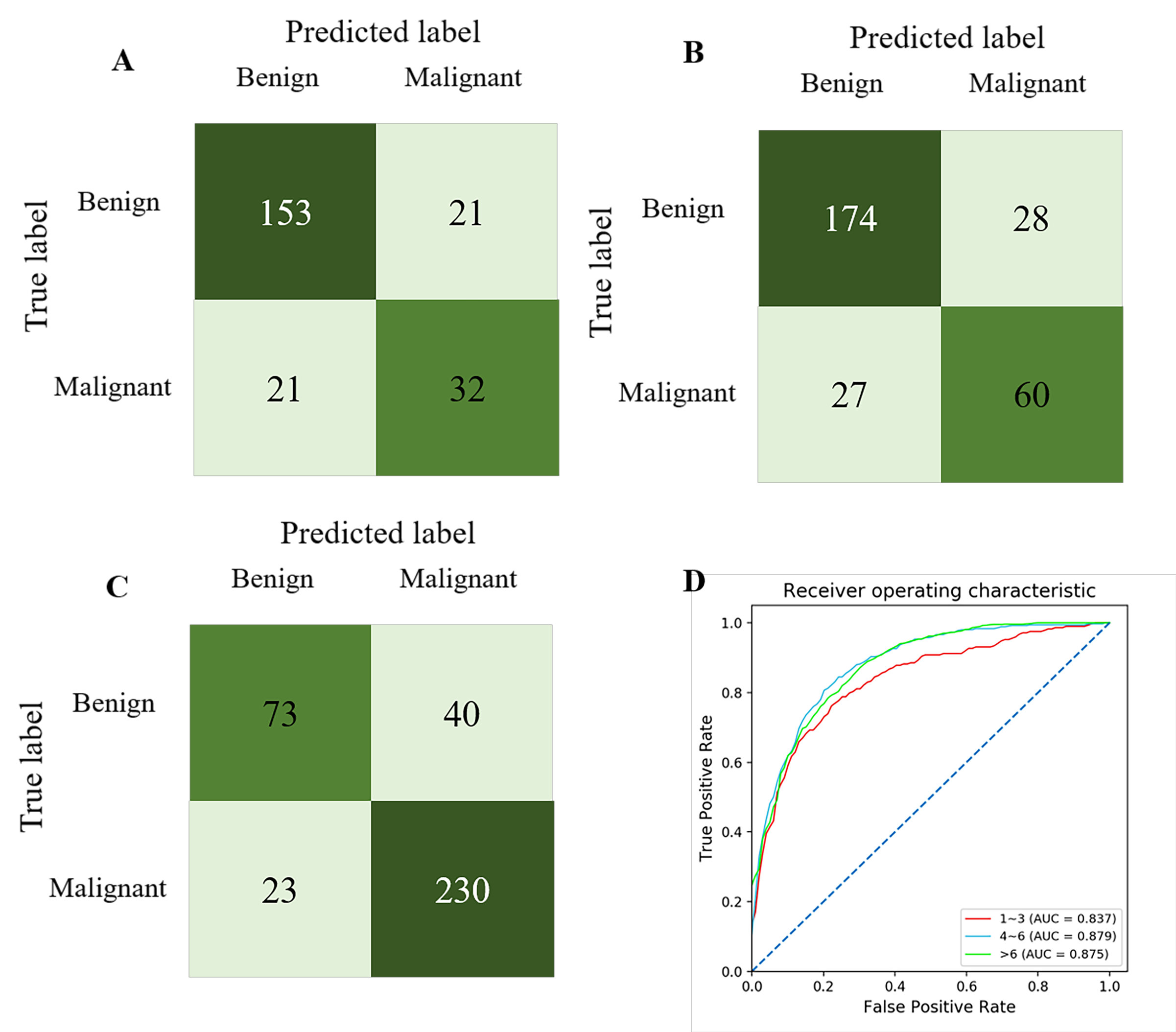

Supplement: Supplementary file 4 — Additional file 4: Fig. S3. The confusion matrix of few lesions group (A), medium lesions group (B), extensive lesions group (C) in breast cancer group. The ROC of the three groups in the lesion-based diagnosis (D). [file 12880_2021_662_MOESM4_ESM.tif]
